# Janus Separator of Polypropylene‐Supported Cellular Graphene Framework for Sulfur Cathodes with High Utilization in Lithium–Sulfur Batteries

**DOI:** 10.1002/advs.201500268

**Published:** 2015-10-01

**Authors:** Hong‐Jie Peng, Dai‐Wei Wang, Jia‐Qi Huang, Xin‐Bing Cheng, Zhe Yuan, Fei Wei, Qiang Zhang

**Affiliations:** ^1^ Beijing Key Laboratory of Green Chemical Reaction Engineering and TechnologyDepartment of Chemical EngineeringTsinghua UniversityBeijing100084P.R. China

**Keywords:** graphene, Janus structure, lithium–sulfur battery, mesoporous carbon, separator

## Abstract

Owing to the conversion chemistry of the sulfur cathode, the lithium–sulfur (Li–S) batteries exhibit high theoretical energy density. However, the intrinsic mobile redox centers during the sulfur/Li_2_S‐to‐lithium polysulfides solid‐to‐liquid phase transition induce low sulfur utilization and poor cycling life. Herein, the Janus separator of mesoporous cellular graphene framework (CGF)/polypropylene membrane to promote the utilization of sulfur cathode is introduced. The porous polypropylene membrane serves as an insulating substrate in contact with lithium anode while CGFs that possess high electrical conductivity of 100 S cm^−1^, a large mesopore volume of 3.1 cm^3^ g^−1^, and a huge surface area of 2120 m^2^ g^−1^ are adhered on cathode side to reactivate the shuttling‐back polysulfides and to preserve the ion channels. Therefore, the Li–S cell with the “two‐face” CGF Janus separator exhibit a high initial capacity of 1109 mAh g^−1^ and superior capacity preserved upon 800 mAh g^−1^ after 250 cycles at 0.2 C, which is 40% higher on sulfur utilization efficiency than the corresponding results with routine polypropylene separators. There are significant improvements on capacity as well as electrochemical kinetics. A very high areal capacity of 5.5 mAh cm^−2^ combined with high sulfur content of 80% and areal loading amount of 5.3 mg cm^−2^ is achieved for such advanced configuration. The negative impact of shuttle mechanism on lowering the utilization of sulfur and overall energy density of a Li–S battery is well eliminated by applying CGF separators. Consequently, employing carbonaceous materials as Janus face of separators enlightens new opportunities for improving the utilization of active materials and energy density of devices that involve complex phase evolution and conversion electrochemistry.

## 1 Introduction

Electrochemical energy storage devices (EESDs, e.g., rechargeable batteries, flow batteries, fuel cells, and supercapacitors) have been widely exploited and rapidly propelled for a low‐carbon, green, and sustainable society. Separator is a crucial component of EESDs and its unique functionalities are indispensable.^1^ For example, separators for secondary batteries and supercapacitors separate the cathode and anode to prevent shorting; while in flow batteries and fuel cells, an ideal separator should selectively control the mass transportation in the cell. But in newly emerging EESDs with revolutionary conversion electrochemistry such as lithium–sulfur (Li–S) batteries and lithium–air batteries, separators are supposed to play a crucial role to fully demonstrate the superior high energy density.

Li–S batteries, employing earth‐abundant, cost‐effective, and environmentally friendly sulfur as the cathode material that exhibits a high theoretical cathode capacity of 1675 mAh g^−1^, have been promised high energy densities of 2600 Wh kg^−1^ theoretically and 500–700 Wh kg^−1^ potentially at low costs. Therefore, Li–S batteries are of paramount interests for both academic and industrial communities.^2^, ^3^, ^4^ However, in sharp contrast to rechargeable lithium‐ion batteries with insertion/extraction mechanism, Li–S batteries have highly mobile redox centers during the sulfur/Li_2_S‐to‐lithium polysulfides (LiPSs) and solid‐to‐liquid phase transition. With the redox materials migrating out of cathode scaffolds and being fixed on lithium anode in solid form, the capacity loses drastically. This is what typical shuttle mechanism describes. Enormous efforts have been dedicated to overcome the shuttle issue, most of which focused on cathode, including (1) designing nanostructured conductive carbon^5^ or polymer scaffold^6^ to confine LiPSs, (2) employing inorganic yet conductive materials for enhancing the adsorption and surface redox chemistry of LiPSs,^7^ and (3) tailoring reduction pathway and chemical formulation of polysulfide complex by tuning the coordination capability of electrolyte solvents.^8^ Although previous works have made huge success, the dissolution of LiPSs seems to be barely evitable in conventional ether‐based liquid electrolytes.

Apart from the electrochemical instability of Li–S systems, the energy density is also unsatisfactory and far below theoretical value.^3^, ^9^ One solution is to increase the content^10^ and the areal loading amount^11^ of sulfur in the cell. But the utilization of active materials usually drops as the amount of sulfur increases.^12^ Another route is to improve the utilization efficiency of sulfur especially at high loading amounts. Actually, the shuttle of LiPSs may bring negative effects more than their parasitic reactions and irreversible loss. First, LiPSs that shuttle back could not be fully utilized due to the interception of porous separators and the passivation of separator/cathode interfaces. Second, the extraction of solid sulfur from the cathode to the electrolyte via dissolution of LiPSs can lead to severer structural collapse than hypothetical solid state transformation from sulfur to Li_2_S with volume expansion of ≈170%. Obviously, shuttle phenomenon not only lowers the service life of Li–S batteries but also impedes the full demonstration of the high energy density. Therefore, utilizing LiPSs that shuttle back at high efficiency, as well as controlling their deposition at electrode/separator interface, should be a key issue.

Recently, engineering functional separators in Li–S batteries is considered as an alternative route to tackle the shuttle issue besides modifying electrode materials and electrolytes.^2^ One effective way is to prevent the diffusion of LiPSs across the porous separator by introducing functional groups as coating layers on the cathode side of separators.^13^, ^14^ For example, our group developed Nafion‐coated^13^ and graphene‐oxide‐coated separators^15^ as ion‐selective membranes to reject LiPSs by electrostatic repulsion. As a result, the cycling stability and Coulombic efficiency have been significantly improved. Another way is to employ solid electrolyte membrane to completely block the permeation of LiPSs.^16^ However, these two ways sacrificed the ion conductivity and the high‐rate performance. Manthiram and co‐workers developed series of carbonaceous interlayers (microporous carbon (MPC) paper,^17^ carbonized natural leaves^18^ and eggshell membranes,^19^ and polyethylene‐glycol‐supported MPC,^20^ etc.) to facilitate the performance of Li–S batteries. The rich micropores provided strong adsorption to LiPSs while the conductive nature of carbon reduced impedances, which led to enhanced utilization of sulfur, prolonged cycling performance, and anti‐self‐discharge capability as well. Zhou et al. coated large‐area graphene flakes on polypropylene (PP) substrates as integrated separator/electrode systems or sandwiched sulfur cathode between two graphene layers to retard the diffusion of LiPSs and to enhance the adhesion of sulfur cathode.^21^, ^22^ The improvement on cycling and rate performance was also pronounced. Other nonporous carbonaceous materials such as carbon black^23^ and carbon nanotubes^24^ also proved concepts of interlayers or carbon‐coated separators. But the main purpose of MPC and nonporous carbon is still to retard the diffusion of LiPSs as physical barriers. Their efficiencies highly depend on the porous structure. However, the pore volume of MPC and carbon black is usually low, impeding the further utilization of large amounts of LiPSs and the permeation and wetting of electrolytes as well. In this regard, mesoporous carbon with high pore volume and large electrochemically active surface area has rarely been reported for functionalizing separators in Li–S batteries.^25^

**Figure 1:**
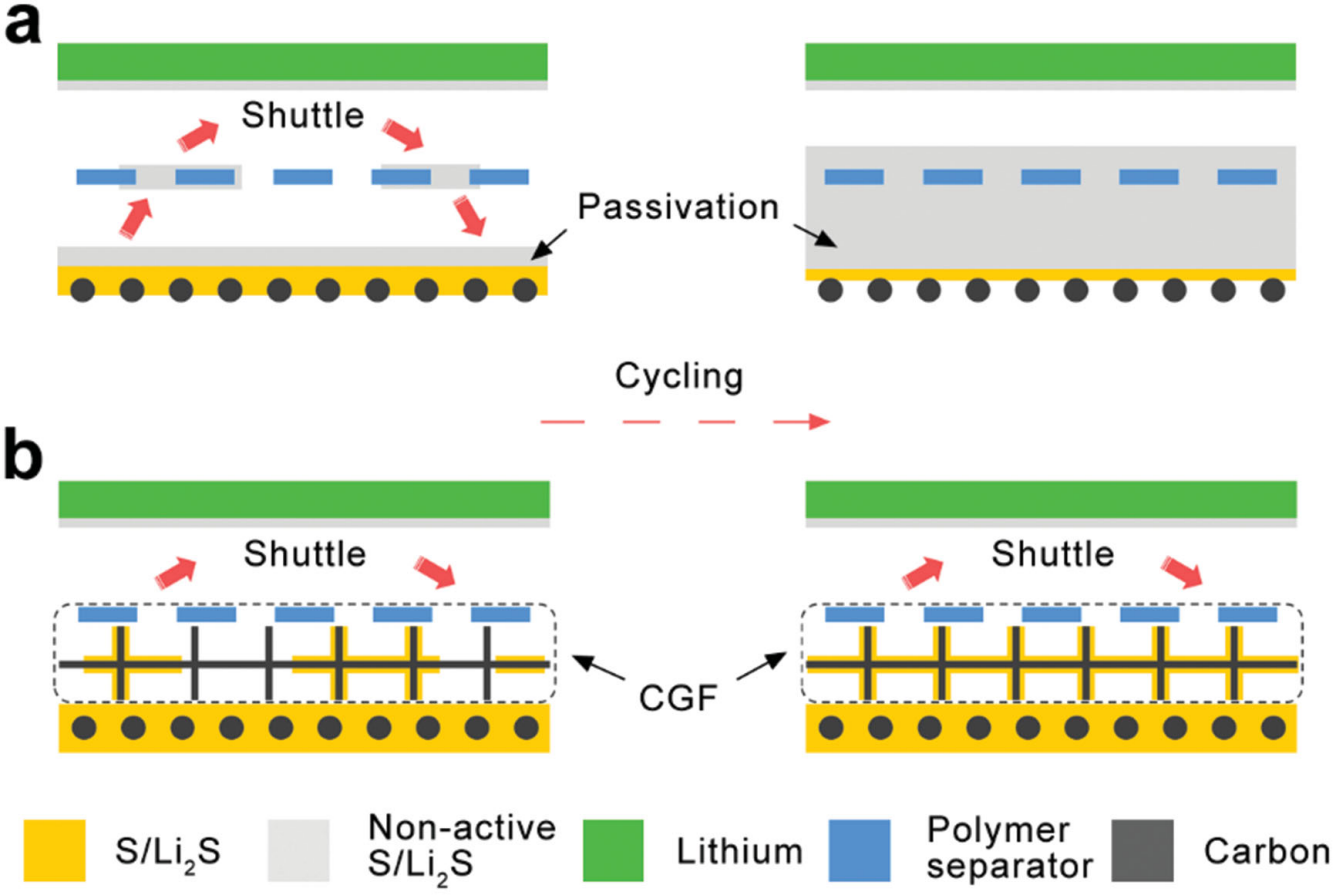
Schematic illustration of a) a routine PP separator and b) a Janus separator with a CGF layer. Without a CGF layer, the LiPSs that shuttled back accumulated at the separator/cathode interface to form a solid, nonactive, insulating passivation layer. With a CGF layer, the LiPSs were reduced and deposited on the conductive scaffolds, preventing the formation of the insulating film and enabling the further penetration of LiPSs into the cathode.

Typically when we consider the interaction between routine porous separators and LiPSs, the shuttle phenomenon should be more complicated (**Figure** **1**a). As the LiPSs diffuse out of the cathode, they shall be easily intercepted by porous separators and irreversibly consumed at the anode through spontaneous side reactions with lithium due to their high mobility and instability. As the LiPSs shuttle back to the cathode/separator interface, they are preferentially reduced at the surface of cathode and form a dense, inert, and insoluble layer, which passivates the conductive surface of the cathode and prevents further reduction of LiPSs. Large amount of sulfur thereby fails to be utilized. After prolonged cycling, the passivation layer evolves to denser and thicker monolith, leading to deteriorative performance. Obviously, routine polymer separator is unable to activate the intercepted and passivated LiPSs because it is electrical insulating. Here, we propose a Janus separator because Janus structures can offer asymmetry and realize the emergence of properties inconceivable for homogeneous or symmetric structures, where the name Janus was derived from a Roman God.^26^ In this Janus separator, nanoporous PP membrane still serves as an insulating substrate in contact with lithium anode while a layer of cellular graphene framework (CGF), which has extraordinary electrical conductivity, abundant in‐plane mesopores, high electrochemical active surface area, and large mesopore volume, adheres to the cathode side to reactivate the shuttling‐back LiPSs and to preserve the ion channels (Figure 1b). The Janus separator of PP‐supported CGF layer (denoted as CGF separator) promises the efficient utilization of sulfur cathode with high capacity and good cycling stability. Moreover, the Janus separator, besides modifications on electrode materials and electrolyte formulations, opens new opportunities for facilitating utilization of active materials that are highly mobile in emerging high‐energy‐density EESDs and proposes a better way to rationally adopt superior characteristics of various novel nanostructured carbon to EESDs.

## 2 Results and Discussion

### 2.1 Morphology and Pore Structure of the Janus Separator with CGF Layer

**Figure 2:**
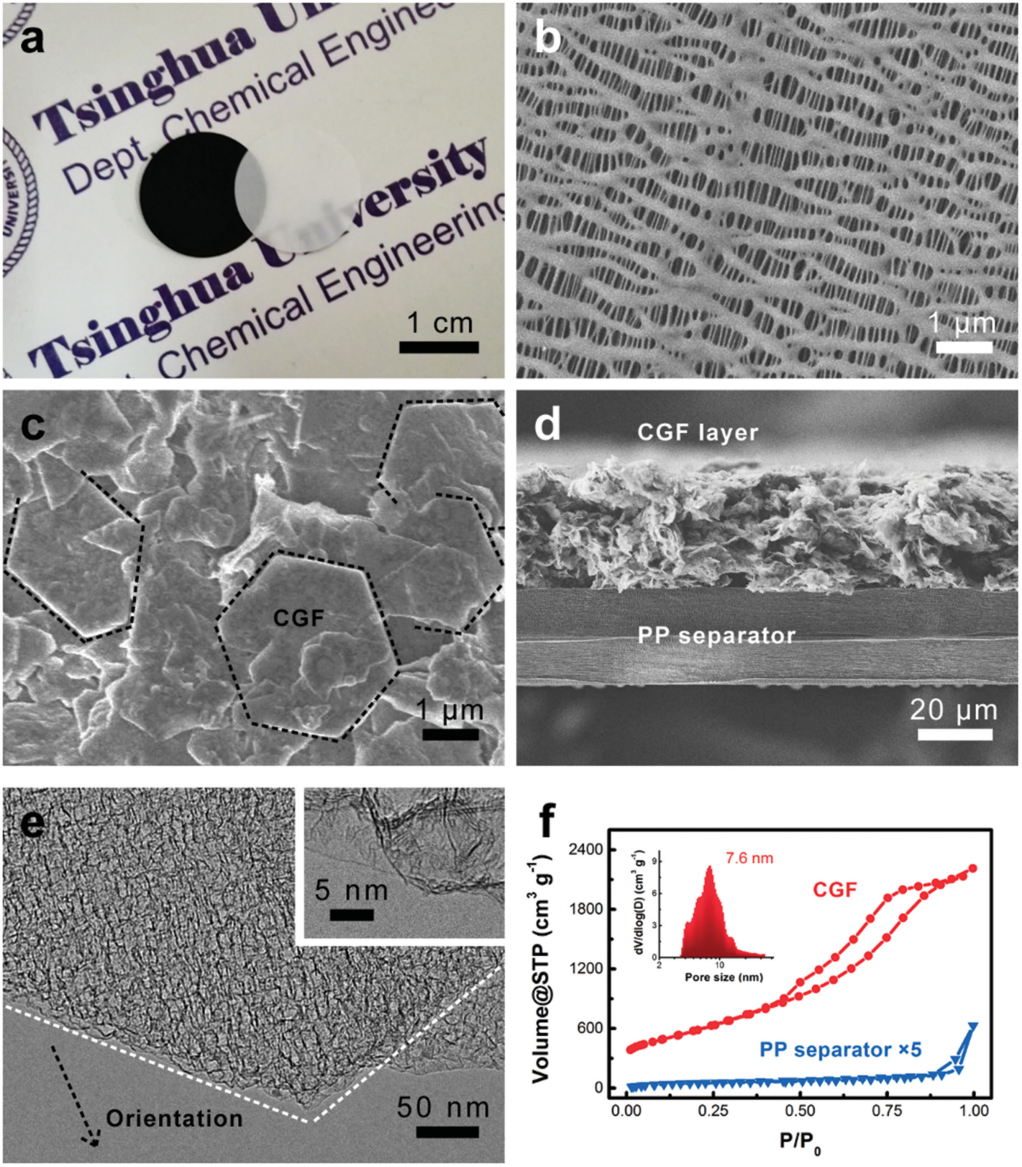
Characterization of Janus separator: a) digital image of white PP separator and black Janus separator with CGF layer (CGF separator); SEM images of b) PP separator, c) CGF separator, and d) cross section of CGF separator showing the Janus structure; e) TEM image of CGF and HR‐TEM image (inset) of a single cellular unit in CGF; f) N_2_ isotherm of PP separator and CGF.

CGFs were firstly obtained by chemical vapor deposition growth of graphene layers on MgO templates, which were calcined from hydrothermally synthesized Mg(OH)_2_ precursors, and subsequent removal of templates.^27^ Then, CGFs were filtrated on PP substrates to form Janus separators. The surface of white PP membrane was thereby coated by a black CGF thin film with a loading amount of 0.3 mg cm^−2^ (**Figure** **2**a). The pristine PP membrane exhibited highly nanoporous polymer matrix with abundant slit pores of around several hundreds of nanometers (Figure 2b), while the CGF layer completely concealed the underneath porous structure of the PP substrate by overlapped hexagonal CGF flakes with lateral size of several micrometers (Figure 2c). The Janus structure was further revealed by the cross‐sectional scanning electron microscopy (SEM) image, where 30 μm thick CGF layer tightly attached on the PP substrate (Figure 2d). The interplane pores of CGFs can be clearly observed, which facilitated the transportation of lithium ions and shuttling‐back LiPSs. Thus, no additional impedance was expected to be rendered by the CGF layer. As shown in Figure 2e, a CGF flake well inherited the hexagonal morphology of Mg(OH)_2_ precursors and as‐calcined MgO templates. After the removal of oxide template, the deposited ultrathin graphene layers interconnected as a uniform, tightly packed, and quasi‐orientated cellular‐like framework. A single nanocellular unit with size around 8–10 nm was further indicated by high‐resolution transmission electron microscopy (HR‐TEM) images (inset of Figure 2e). The nanocellular unit was mainly wrapped by 1–2 layers of graphene. The highly interconnected graphene cells with intact sp^2^‐carbon junctions rendered the whole CGF with extraordinary electrical conductivity of 100 S cm^−1^, which was obtained by four‐probe electrical test for CGF pellets made of compressed powders. Due to the template effect of tightly packed MgO nanoparticles, the ultrathin graphene layers were highly curved, which thereby eliminated their packing tendency and remained the large interior cavity. As a result, the conductive surface was fully exposed and electrochemically accessible while the available pore volume of mesoporous nanocellular framework was boosted, both of which were expected to be beneficial for electrochemical performance. Quantitative analysis on pore structure was interpreted by N_2_ isothermal adsorption (Figure 2f). CGFs with thin and light graphene‐like cellular structure got the ascendance to PP membranes on adsorptive capability at relative pressure of 0.05–0.90, where the adsorption of mesopores was predominant. As the density functional theory (DFT) calculation illustrated, the average mesopore size was 7.6 nm while the mesopore volume was extraordinarily high as 3.1 cm^3^ g^−1^ (inset of Figure 2f). The overall specific surface areas resolved by Brunauer–Emmett–Teller (BET) theory and DFT method were both around 2120 m^2^ g^−1^, 80% of which was contributed by cellular‐like mesopores as DFT results indicated. Therefore, the CGF has comparable surface area to MPCs but the available pore volume and electrochemical active surface area were much higher because the dominant mesopores are more ion‐accessible than tortuous micropores. These unique characteristics of CGF rendered as‐prepared Janus separators with superior performance. In contrast to CGF, nonporous SuperP only had a low specific surface area of 63 m^2^ g^−1^ and a pore volume of 0.16 cm^3^ g^−1^, which was applied as controlled samples to coat PP separators with the same areal loading amount of carbon (0.3 mg cm^−2^) (Figure S1, Supporting Information). The thickness of SuperP‐coating layer was ≈10 μm, a third that of the CGF layer. There was significant gap between SuperP separators and CGF separators in capability of accommodating and utilizing LiPSs. The uptake of liquid electrolyte was thereby tested as 170%, 168%, and 220% for PP separators, SuperP separators, and CGF separators, respectively (Figure S2, Supporting Information). The remarkably high pore volume and contact surface of CGF accounted for far exceeding uptake of electrolyte (457%) than PP (170%) and SuperP (157%) materials. Therefore, the Janus‐type CGF separators with abundant mesopores and conductive scaffolds are highly desirable as reaction chambers to reutilize the shuttling‐back LiPSs.

### 2.2 Electrochemical Performance of Li–S Batteries with CGF Separator

**Figure 3:**
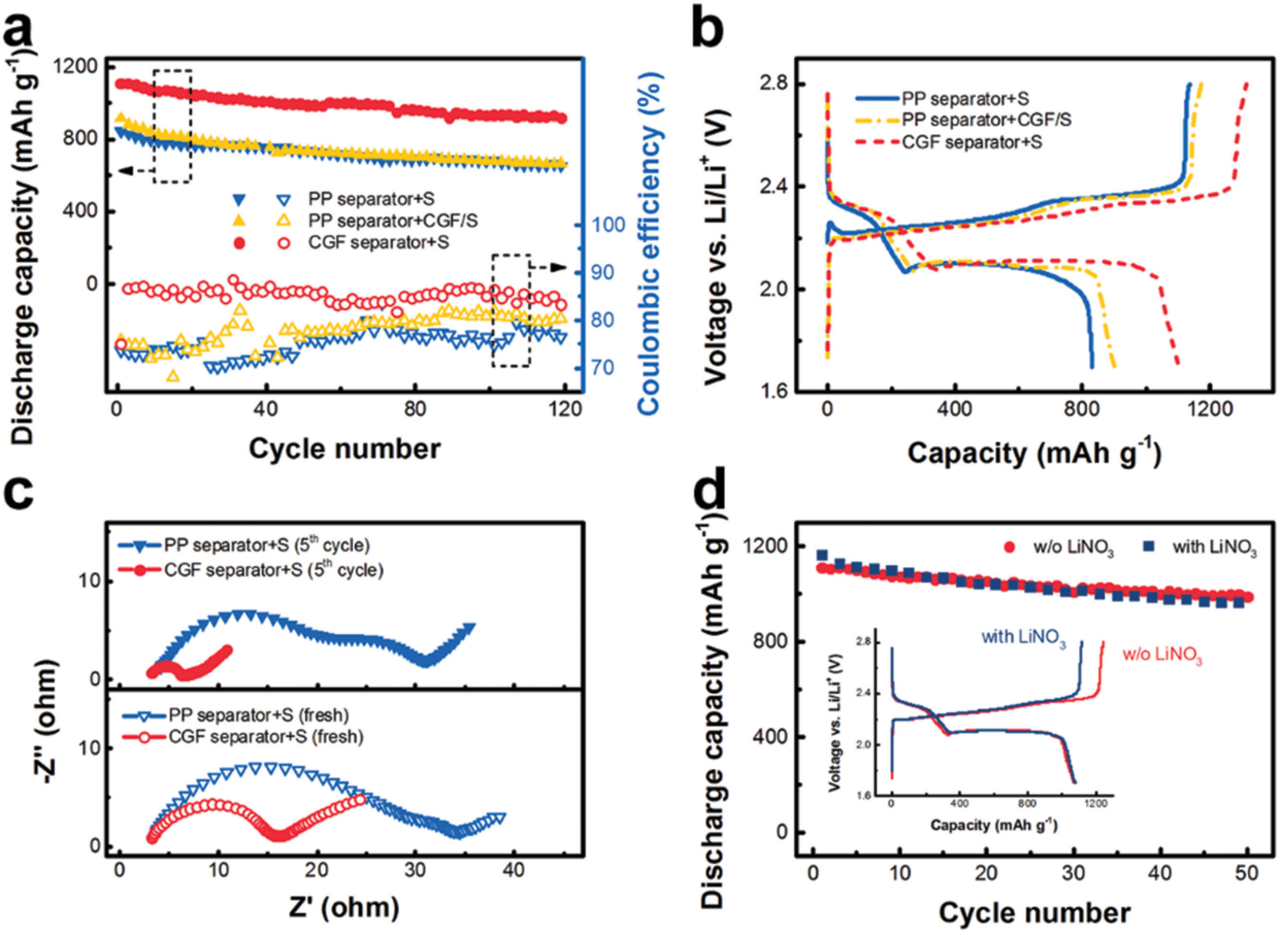
Electrochemical performance of Li–S batteries with CGF separator: a) cycling performance and b) corresponding galvanostatic discharge‐charge curves at 0.2 C; c) Nyquist plots of fresh cells and cells cycled for 5 cycles; d) cycling behaviors of Li–S batteries with CGF separator with and w/o LiNO_3_.

A pronounced improvement in electrochemical performance of Li–S batteries was achieved by employing Janus‐type CGF separators. As shown in **Figure** **3**a, the initial capacity of the sulfur cathode was significantly facilitated by the CGF separator from 846 to 1109 mAh g^−1^ at a current density of 0.2 C (1.0 C = 1675 mA g^−1^, according to the mass of sulfur), corresponding to an increase over 30% in utilization efficiency of sulfur. Moreover, the capacity of the sulfur cathode utilizing a CGF separator remained at 915 mAh g^−1^ after 120 cycles, which was almost 40% higher than its counterpart with a PP separator. After even longer operation at a low rate of 0.2 C over 250 cycles, a stable and reversible capacity over 800 mAh g^−1^ can still be retained (Figure S3, Supporting Information). Apparently, replacing routine PP membrane into a well‐designed Janus separator can significantly promote the utilization of sulfur cathodes and the cycling stability as well. But when the pristine sulfur cathode was mixed with the same amount of CGFs as in the case of a CGF separator coupled with a bare sulfur cathode, the battery performance was not that remarkable. There was only 8% of enhancement on initial capacity to the sulfur cathode, which might be aroused from the higher conductivity and surface area of CGF than SuperP. However, even such a minor improvement was still offset during the following 20 cycles, and the CGF‐blended sulfur cathode showed no competitiveness to the sulfur cathode in the following 100 cycles. Note that the employment of CGF separator was not a trick to add more carbon in the system since the sulfur‐to‐carbon ratio was delicately controlled same as 1.4 in all cells whatever cathodes or separators were tested. Therefore, pasting CGFs on polymeric substrates to construct Janus separators is a better way to demonstrate the structural advantages of CGFs than directly applying them in the cathode. This principle can also guide the application of other novel nanostructured carbon materials.

The huge promotion was deliberately attributed to the activation effect of the CGF layer on interfacial sulfur compounds that accumulated between the insulating but porous PP ­membrane and the sulfur cathode. First, the Coulombic efficiency of the sulfur cathode with a CGF separator was still lower than 90% though slightly higher than the cathode with a PP separator (Figure 3a). It suggested that CGFs can afford comparable confinement for LiPSs to neither MPCs^17^, ^18^, ^19^, ^20^ nor Nafion/graphene oxides^13^, ^15^ because of the large size of the mesopores. Therefore, the physical interception of the CGF layer to prevent the diffusion of LiPSs across separators should be of minor dominance. Second, the improvement on capacity as well as electrochemical kinetics was more pronounced than simple inhibition of shuttling across membranes (Figure 3b). Note that there was an obvious voltage drop at the lower discharge plateau for PP separator/sulfur cathode while CGF separator did not display such deterioration. That meant that more energy was consumed for the kinetically sluggish conversion from liquid LiPSs to solid Li_2_S/Li_2_S_2_ without CGF layers. The overpotential at the beginning of charging was also suppressed by CGF separators, suggesting the reduced domain size of insulating Li_2_S phase. Electrochemical impedance spectra further verified the enhanced kinetics of Li–S batteries with CGF separators compared to those with pristine PP separators (Figure 3c). The resistance of charge transfer (*R*_ct_, indicated by the semicircle located at the middle frequency) of fresh cell with CGF separators was around 50% less than that with PP separators, which was aroused from the superior conductivity of CGF (100 S cm^−1^). More notably, *R*_ct_ of cells with CGF separators ­significantly dropped to less than 20% of cells with PP ­separators after five cycles. The decrease on *R*_ct_ was attributed to redistribution of active materials in CGF layer and cathode induced by shuttle phenomenon. However, for cycled cells with PP separators, not only the *R*_ct_ changed little but also an additional semicircle appeared at lower frequency, indicating that a new resistive phase was formed with only PP membranes applied. Third, whether the lithium metal anode was protected did not leave a strong impact on battery performance, but whether the CGF layer was adopted virtually did. The addition of lithium nitrate that was commonly used to passivate lithium anode^28^ rendered almost no change in capacity and its retention except for reduced overcharging (Figure 3d). Even though the anode was well protected, the sulfur cathode barely with PP separator could not be comparable to cells made of unprotected lithium/CGF separator/sulfur cathode (Figure S4, Supporting Information). It suggested that at least in coin cell level, irreversible loss of LiPSs through spontaneous reactions with fresh lithium was not a major route to lose active phases and the capacity.

### 2.3 Postmortem Analysis on Cycled Batteries for Investigating the Activation Effect of CGF Separators

**Figure 4:**
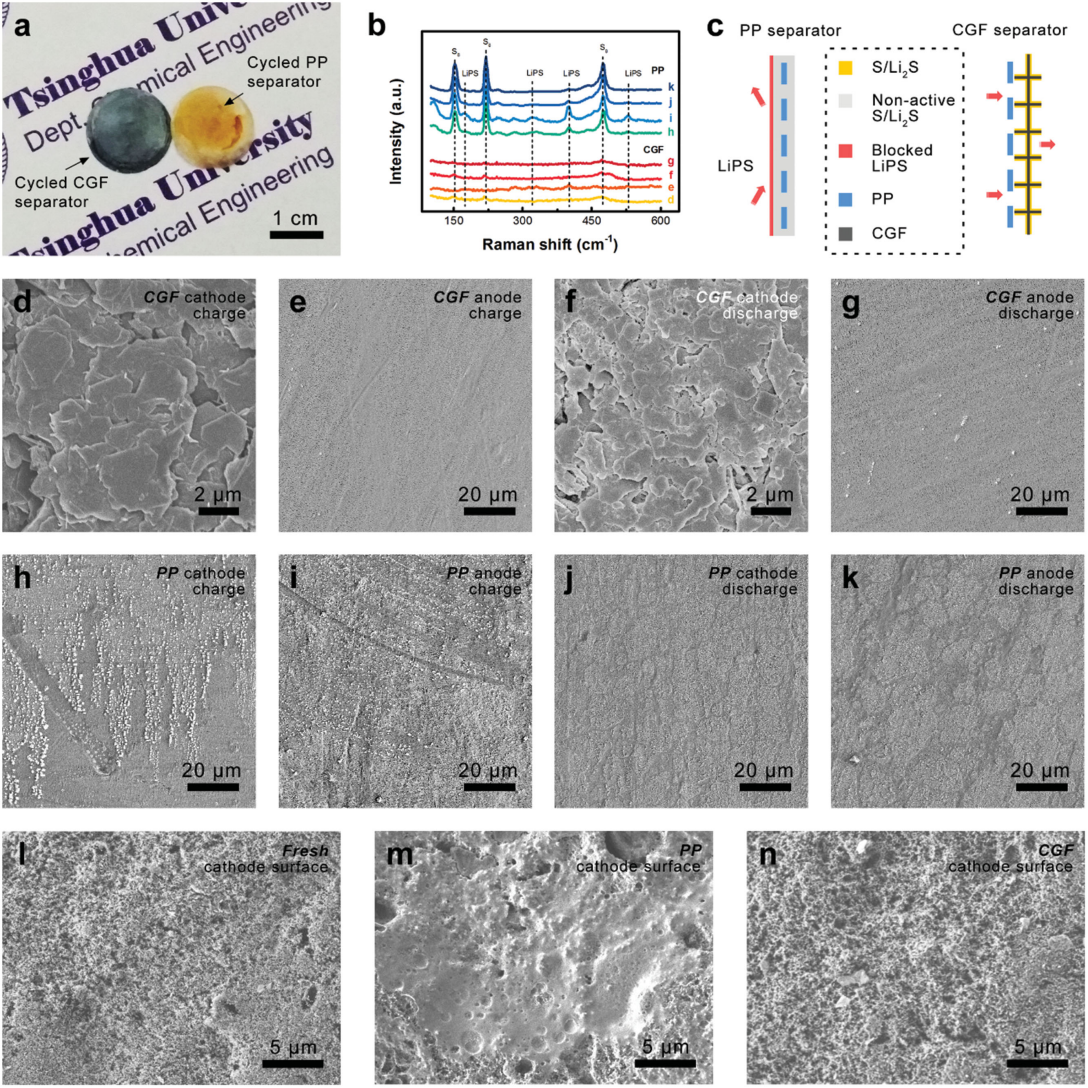
Morphology and composition of cycled separators and cathodes: a) digital image of a cycled PP separator and CGF separator; b) Raman spectra of cycled PP separators and CGF separators at different states as indicated in (d–k); c) Schematic illustration of cycled PP separators where dense, nonactive film formed and CGF separators with unblocked ion channels; SEM images of cycled CGF separators: d) cathode and e) anode sides at charged state, f) cathode and g) anode sides at discharged state; SEM images of cycled PP separators: h) cathode and i) anode sides at charged state, j) cathode and k) anode sides at discharged state; SEM images showing surfaces of l) pristine cathode, m) cycled cathode with PP separator, and n) cycled cathode with CGF separator. The separators in (d–k) were cycled for 5 cycles whereas the cathodes in (m, n) were cycled for 50 cycles.

The hypothesis that the CGF layer activates shuttling‐back LiPSs back was elaborately investigated by postmortem analysis on cycled batteries (**Figure** **4**). Dark yellow precipitates were observed on the surface of cycled PP separator on the anode side, visibly indicating the interception of sulfur species by porous polymer matrix (Figure 4a). However, CGFs tightly adhering to the PP substrate can ignite the electrochemical activity of intercepted sulfur compounds when they diffused back and contacted with the full‐coverage conductive scaffold of CGF. Thus, the CGF separator showed much lighter yellow surface. Even after long cycling, the CGF layer still tightly attached to the PP substrate (Figure S6, Supporting Information). Therefore, there is no problem with the adhesion property between PP and CGF. The chemical composition of the yellow precipitation was further validated by ex‐situ Raman spectroscopy (Figure 4b). Surface compositions of cycled CGF separators of cathode/anode sides at charged (2.8 V)/discharged (1.7 V) states (denoted as d–g in Figure 4b, corresponding to the SEM images of Figure 4d–g, respectively), as well as cycled PP separators of the same status (denoted as h–k in Figure 4, corresponding to the SEM images of Figure 4h–k, respectively), were obtained. All the disassembled cells were cycled for 5 cycles. Three major peaks at 152, 218, and 470 cm^−1^ were assigned to elemental sulfur while LiPSs with various formulas accounted for other predominant resonances.^29^ The cycled PP separators of each status (h–k in Figure 4) exhibited much more remarkable emergence of sulfur and LiPSs than cycled CGF separators (d–g in Figure 4). Note that even at fully discharged state (1.7 V), the existence of sulfur in/on PP separators can still be clearly verified (j and k in Figure 4). The comparison between fully charged states (h and i in Figure 4) and discharged states (j and k in Figure 4) of cycled separators further indicated that LiPSs could not be easily charged back to sulfur as the strong resonances of high‐order LiPSs at ≈400 cm^−1^ for charged PP separators indicated.^30^ Moreover, LiPSs tended to accumulate more at the anode side of PP separators than the cathode side after charging to 2.8 V (h and i in Figure 4), suggesting that LiPSs indeed shuttled to the anode side and were repelled to shuttle back through the PP membranes by insoluble sulfur‐containing deposits. In contrast, the as‐described negative impact on utilizing shuttling LiPSs was thoroughly eliminated by CGF separators since neither the signals of sulfur were strong nor were the LiPSs (d–g in Figure 4). The energy dispersive spectroscopic results further proved that more sulfur species would reside in/on PP membranes without CGF Janus face (Figure S5, Supporting Information). Therefore, sulfur and LiPSs were suggested to accumulate in/on the porous PP separators instead of being utilized as in/on CGF separators (Figure 4c). As cycling being more prolonged, the accumulated, nonactive and insoluble film would further deteriorate the mass transport in the cell and the whole battery performance as well because of the impermeable and insulating nature of sulfur‐containing deposits. However, the Janus face of CGF can activate the intercepted sulfur compounds and accommodate them without blocking the ion channels. The large pore volume, high surface area of mesopores, and the extraordinary electrical conductivity ensured the sustainable positive functions of CGF layers for Li–S batteries with long service life.

The morphology of cycled separators and electrodes was thereby distinguishable. After 5 cycles, the upper surface of CGF separators at both charged and discharged states preserved the porous structure of overlapped CGF flakes (Figure 4d,f). No large aggregation of solid sulfur compounds can be detected. Even after 50 cycles, the hexagonal shape of CGF flakes was still unconcealed by the solid deposits while the porous structure was also retained (Figure S7a, Supporting Information). Little visible solid particle could be observed at the anode side of cycled CGF separators as well (Figure 4e,g). The surface was very clean with still identifiable slit pores of PP substrates. However, for the cycled PP separators at charged state, a great number of particles was observed at each sides of PP separators (Figure 4h,i). The particle size was around 200 nm to 1 μm at the cathode side while it was smaller at the anode side (Figure S8a,b, Supporting Information). But for both cathode side and anode side, the slit pores of underlying PP substrates were less visible than fresh PP membranes, indicating the blocked porous structure of PP membranes after cycling (Figure 2b). While for the PP separators at discharged state, a large proportion of the surface was penetrated by insoluble compounds, exhibiting spreading “landscapes” of white stripes or patches in Figure 4j,k. The magnification of these “white patches” showed mostly clogged pores of PP substrates (Figure S8c, Supportiong Information). After 50 cycles, the relatively small solid deposits evolved into rampant and larger aggregates, fully occupying the surface of PP membranes and thereby inhibiting the mass transportation (Figure S7b, Supporting Information). The engineered Janus face of CGF also left a pronounced impact on the morphology of the cathode. The fresh cathode was composed of blended sulfur and carbon particles with abundant pores (Figure 4l). However, after 50 cycles with the PP separator, the surface of the cathode was covered by a dense, smooth, and impervious film, which was mainly composed of nonactive solid sulfur species as discussed above (Figure 4m). The accumulation and passivation of sulfur compounds at the PP separator/cathode interface instead of further penetration into the carbon scaffolds in the cathode led to low utilization and poor stability of sulfur‐involved conversion chemistry. However, in terms of the activation effect of the CGF separator, the formation of an inert film at the separator/cathode interface was restrained and the porous structure of sulfur cathode was conserved as well (Figure 4n). Therefore, a better performance of Li–S batteries can be expected by applying CGF separators.

### 2.4 Advanced Li–S Batteries Based on Sulfur Cathode Enabled by CGF Separators

**Figure 5:**
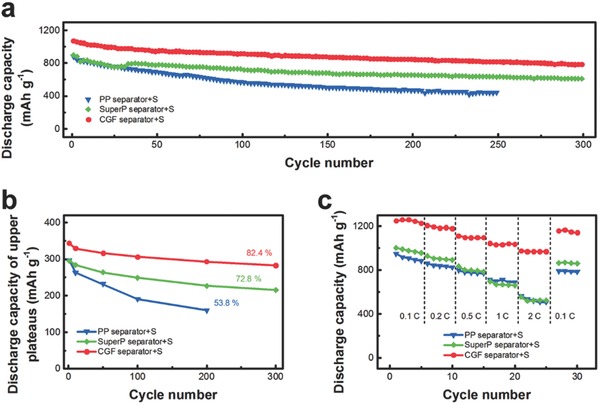
Long‐term cycling performance and rate capability of Li–S batteries with CGF separator: a) cycling performance and b) corresponding discharge capacity of upper plateaus at 0.5 C; c) rate performance.

Both the highly desirable mesopore‐dominated architecture and a three‐dimensionally extending graphene framework are the key structural features of CGFs to compete against other nonporous carbonaceous materials for a Janus‐type separator. Especially at higher current densities, the superiority of CGF separators was more compelling (**Figure** **5**). When cycled at a high rate of 0.5 C, the cycling stability of the sulfur cathode with a PP separator was even worse than that at 0.2 C (Figure 5a). Though applying high current densities can reduce overcharge by shortening the charging time,^13^ the LiPSs that shuttle back are more likely to be reduced to form aforementioned nonactive interphases with an increased current density, as soon as they contact the outmost conductive scaffolds of the cathode. That is owing to their diffusion rate incompatible to the high current density. Thus, the deep penetration of LiPSs is impeded. As a result, the capacity faded very fast and only a capacity of 441 mAh g^−1^ can be attained after 250 cycles at 0.5 C. Employing nonporous SuperP as a Janus face of the separator can enhance the cycling stability to some extent but the initial capacity was still low. The sulfur cathode with a CGF separator possessed the highest initial capacity of 1072 mAh g^−1^ and also a capacity reserved upon 800 mAh g^−1^ after 300 cycles at 0.5 C, which was around 30% and 80% higher than its counterparts with a SuperP separator and a PP separator, respectively. Such a significant improvement on cycling performance by using CGF separators was mainly aroused from the high and reversible utilization of active materials as Figure 5b indicated. The upper plateau in addition with the subsequent slope corresponds to the conversion from sulfur to Li_2_S_4_ with a theoretical capacity of 418 mAh g^−1^. Its capacity indicates how much sulfur is available for electrochemical reduction. The cell with a CGF separator had highest initial capacity of upper plateau and also the highest retention of 82.4% upon 300 cycles. However, the retention was only 53.8% and 72.8% for cells with a PP separator and a SuperP separator, respectively. The CGF separator also fabulously facilitated the rate performance of the sulfur cathode (Figure 5c). The capacity of a CGF separator/sulfur cathode at 0.1 C was 1259 mAh g^−1^ and exhibited a remarkable retention of 975 mAh g^−1^ at 2.0 C, which was 73% higher than that of a PP separator/sulfur cathode. Except for the higher capacity, the polarization at high rate was also reduced by adopting a CGF separator (Figure S9, Supporting Information). The polarization voltage was 383 mV at 2.0 C for the cell with a CGF separator while that was much larger as 668 and 569 mV for cells with a PP separator and a SuperP separator, respectively. The CGF separator afforded the lowest polarization voltage not only because of the highest electrical conductivity of CGFs but also due to the inhibition of the nonactive separator/cathode interphases at higher current rates. The Janus face of CGF that had high uptake of electrolytes as well as a large pore volume and a high electrochemical active surface area was considered to better utilize the shuttling‐back LiPSs and also to prevent their passivation.

**Figure 6:**
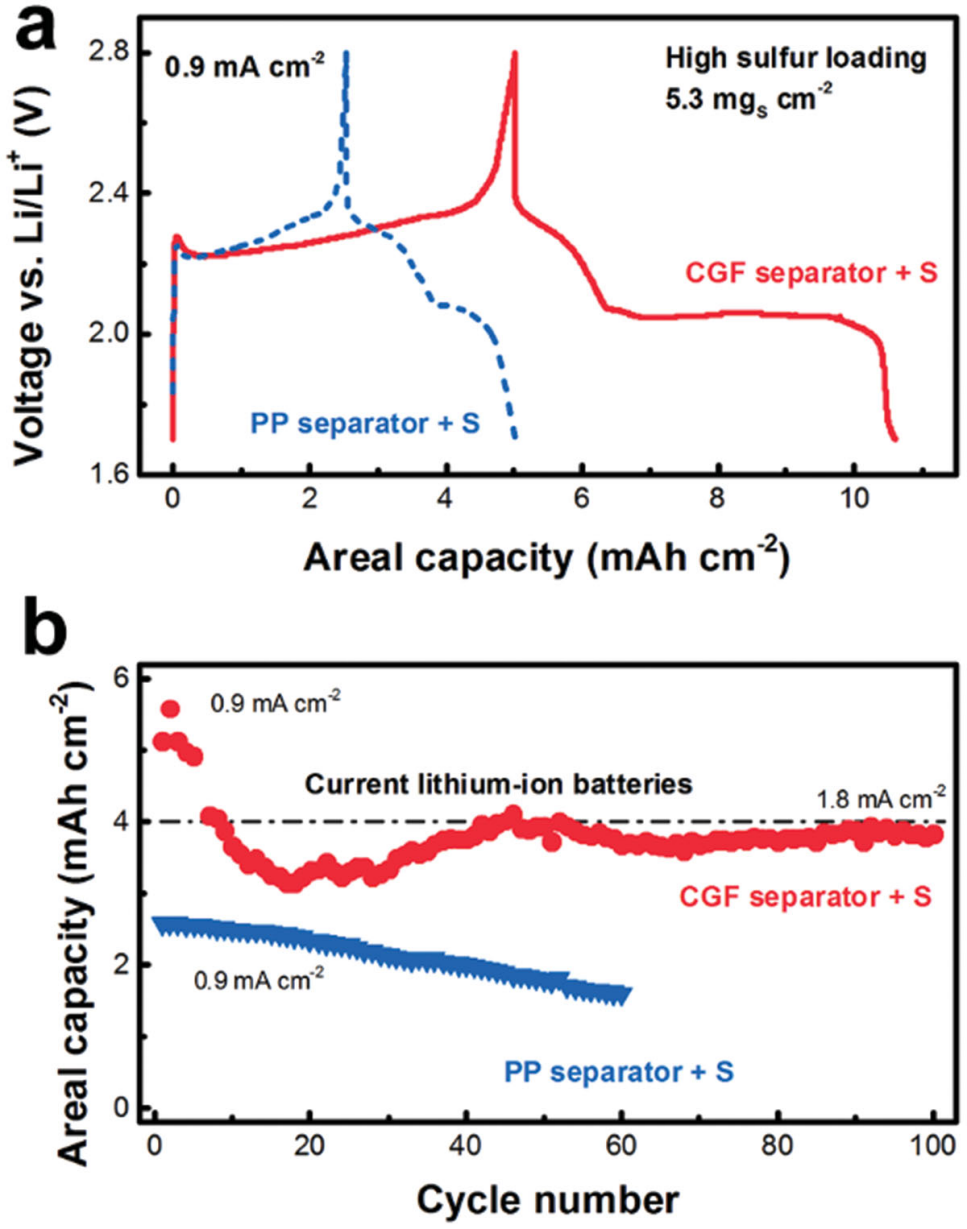
High‐sulfur‐loading Li–S batteries with CGF separator: a) galvanostatic charge–discharge curves at a current density of 0.1 C (0.9 mA cm^−2^) and b) cycling performance at current densities of 0.1 C (0.9 mA cm^−2^) and 0.2 C (1.8 mA cm^−2^).

For further enhancing the energy density of the Li–S cells, the sulfur cathode with high sulfur loading amount of 5.3 mg cm^−2^ and high sulfur content of 80% was prepared and assembled with a CGF separator and a lithium foil as the anode to construct an advanced Li–S battery. An outstanding areal capacity of 5.5 mAh cm^−2^ was obtained at current density of 0.9 mAh cm^−2^ (0.1 C according to the mass of sulfur) (**Figure** **6**a). In contrast, the high‐loading sulfur cathode with PP separator exhibited much inferior capacity and liquid‐to‐solid kinetics. Moreover, the CGF separator substantially improved the cycling stability (Figure 6b). Even after 100 cycles at a high current density of 1.8 mAh cm^−2^, the high‐loading sulfur cathode with a CGF separator delivered an areal capacity of approximately 4 mAh cm^−2^ with very low decay rate of 0.064% per cycle, approaching the practical requirement; while the decay rate for the sulfur cathode with a PP separator was 0.64% per cycle, which is an order of magnitude higher than its counterpart with a CGF separator. To the best of our knowledge, such an enhancement via separator engineering has never been realized previously for Li–S batteries with both high sulfur content and high loading amount. Note that the CGF layer only accounted for 5.6% and 3.9% of the mass of the sulfur and the whole electrode, respectively. The specific capacity based on the mass of the whole electrode and CGF layer was 703 mAh g^−1^, which thereby corresponded to gravimetric energy density of 1472 Wh kg^−1^ based on the integrated electrode/functional CGF layer. While to obtain a higher volumetric energy density, either compressing the CGF separator at a pressure of 10 MPa or increasing the areal sulfur loading amounts were applied. After compression, the thickness of CGF layer decreased from ≈30 to 6 μm, corresponding to a decrease of 120% thickness of PP separator to 24% (Figure S10a, Supporting Information). Such a largely reduced thickness of CGF layer did not strongly hamper the battery performance (Figure S10b, Supporting Information). As a result, an optimal volumetric energy density of 827 Wh L^−1^ can be achieved based on the integrated electrode/functional CGF layer, in which the electrode, the compressed CGF layer, and the PP substrate possess thicknesses of 100, 6, and 25 μm, respectively (Figure S11 and Table S1, Supporting Information). Such a volumetric energy density is comparable to other reported values for lithium–sulfur batteries.^31^ Therefore, applying such an engineered Janus separator of CGF can notably enhance the overall energy density of the Li–S batteries, promising a new way besides modifying the electrodes and electrolytes.

Compared to previous works on separators or interlayers for Li–S batteries, the concept of Janus separator shows its unique attributes. (1) To the best of our knowledge, the employment of mesopore‐dominant graphene‐like materials as functional layers on separators for Li–S batteries was reported for the first time in this contribution. In the family of carbonaceous materials, MPCs in various forms (e.g., MPC paper and its composite,^17^, ^20^ carbonized biomass membranes,^18^, ^19^ and nonporous carbon (e.g., carbon black,^23^ graphene flakes^21^ have demonstrated their effectiveness for coating the separators or serving as interlayers to enhance the performance of Li–S batteries. In terms of the structural advantages of mesoporous carbon including high electrochemically active surface area (≈1700 m^2^ g^−1^ for mesopores of CGFs), large and available pore volume (3.1 cm^3^ g^−1^ for mesopores of CGFs), and sometimes geometrical regularity, in combination with the extraordinary electrical conductivity of sp^2^‐carbon‐linked graphene‐like basal units (100 S cm^−1^ for CGFs), the CGF material ought to exert its great potential as a Janus face of separators for benefiting the electrochemical performance of sulfur cathode. (2) Although the CGF layer with large‐size mesopores is neither as efficient as physical/chemical barriers nor LiPSs traps, the LiPSs diffusing back from the anode can be reactivated as redox materials for reversible storage of lithium ions. Consequently, the sulfur cathode with CGF separators exhibited significantly improved capacity, cycling stability, and rate capability. Such a mechanism for improving the Li–S battery performance is in good accordance with recent findings in membrane‐free Li–S batteries^32^ and conductive‐coated separators.^21^, ^23^, ^24^ (3) Attributed from the high sulfur utilization with CGF separators, the negative impacts of shuttle mechanism on lowering the utilization of sulfur and overall energy density of a Li–S battery, were well eliminated by applying CGF separators but clearly revealed for routine PP separators. In addition, the significance of large pore volume and available surface area was also highlighted by comparing CGF separators with nonporous SuperP‐coated separators (Figure 5), which guaranteed the high utilization of sulfur even at a very high areal loading amount of sulfur. Although pristine SuperP‐coated separators showed abundant interparticle pores, which resemble to previously reported carbon‐coated separators,^23^, ^24^ passivation layer still formed between the separator and sulfur cathode after cycling due to the much smaller pore volume of SuperP (0.16 cm^3^ g^−1^) than that of CGF (Figure S12, Supporting Information). (4) The success of CGFs as a Janus face of the separator rather than as the cathode scaffold indicated a more advanced way to demonstrate the applicable potential of a novel carbon material in Li–S batteries (Figure 3a,b).

## 3 Conclusions

The Janus separator of mesoporous CGF/PP membrane was fabricated and applied for Li–S batteries. CGFs, which possessed an average mesopore size of 7.6 nm, a specific surface area of 2120 m^2^ g^−1^ that was electrochemically accessible, a mesopore volume of 3.1 cm^3^ g^−1^, and extraordinary electrical conductivity of 100 S cm^−1^ enabled the “two‐face” separator a high uptake of liquid electrolyte of 220% and a highly interconnected, porous, and conductive scaffold at the separator/electrode interface. Therefore, LiPSs that diffused back from the anode were not intercepted by or attached to insulating polymer substrate, but were reactivated at conductive Janus face of CGF, preventing the formation of a nonactive, resistive, and insoluble interphase. Consequently, the Li–S cell with a Janus CGF separator and a sulfur cathode exhibited a high initial capacity of 1109 mAh g^−1^ and a capacity remained 800 mAh g^−1^ after 250 cycles, which was 40% higher than the corresponding results of routine PP separators. There were significant improvements on capacity as well as electrochemical kinetics. For advanced configuration with high sulfur content of 80% and sulfur loading of 5.3 mg cm^−2^, a very high areal capacity of 5.5 mAh cm^−2^ was achieved. Furthermore, the negative impacts of shuttle mechanism on lowering the utilization of sulfur and overall energy density of a Li–S battery was well eliminated by applying CGF separators. Consequently, employing carbonaceous materials as the Janus face of separators enlightens new opportunities for improving the utilization of active materials and the energy density of EESDs that involved complex phase evolution and conversion electrochemistry, including Li/Na–S/Se batteries, Li/Na–air batteries, organic lithium batteries, and redox flow batteries, where mobile redox centers easily migrated and detached from electrode surface. Engineering the Janus face with different electron/ion conductivities, hydrophobic/hydrophilic affinities, and hierarchical porous structures could fully activate the migrated or shuttled redox materials and guide the design principles for synthesizing and applying advanced energy materials.

## 4 Experimental Section

*Fabrication of Janus Separator*: The carbonaceous materials were coated on the PP substrates via facile filtration. In a typical procedure, 18.0 mg of carbon (CGF fabricated by a modified template chemical vapor deposition method on hydrothermally synthesized MgO templates^27^ or Super P purchased from TIMCAL Ltd.) and 2.0 mg of poly(vinylidene fluoride) (PVDF) binder were dispersed in *N*‐methyl‐2‐pyrrolidone (NMP) by ultrasonication for 1.0 h. Then, 36.0 mL of the dispersion was filtered through a piece of commercial PP separator (Celgard 2400) and subsequently dried at room temperature for 24.0 h. The Janus separator was with carbon loading amount of 0.3 mg cm^−2^.

*Fabrication of Sulfur Cathode*: Slurry coating method was used to prepare sulfur cathode. The slurry was prepared by mixing commercial sulfur powders, carbon materials, and PVDF binder with desirable ratio in NMP. For routine electrochemical evaluation, the slurry was coated onto aluminum foils using a doctor‐blade, dried at 60 °C for 24.0 h, and punched into disks with diameter of 13 mm. The sulfur mass loading was approximately 1.2 mg cm^−2^ and the sulfur content in the whole cathode, including all the components, in addition with the mass of carbon‐coating layer on the separator was 52%. For high‐sulfur‐loading cathode, the slurry was coated onto a carbon nanotube paper, where the sulfur mass loading was 5.3 mg cm^−2^ and the sulfur content in the slurry was 80%.

*Structural Characterization*: The morphology of the Janus membrane was characterized by a JSM 7401F (JEOL Ltd., Tokyo, Japan) SEM operated at 3.0 kV and a JEM 2010 (JEOL Ltd., Tokyo, Japan) TEM operated at 120.0 kV. The pore‐size distribution and BET specific surface area of the samples were measured by N_2_ isothermal adsorption/desorption using Autosorb‐IQ2‐MP‐C system. The pore size distribution and pore volume of the samples were calculated by the quenched solid state DFT method using adsorption branches. Raman spectra were recorded with He‐Ne laser excitation at 633 nm using Horiba Jobin Yvon LabRAM HR800 Raman Spectrometer. The powder conductivity of CGF was obtained using the KDY‐1 four‐probe technique.

*Electrochemical Evaluation*: The standard 2025 coin‐type cell was employed for tests. The cells were assembled in an Ar‐filled glove box, employing sulfur cathode, lithium foil as anode, and different separators. The electrolyte was 1.0 m lithium bis(trifluoromethanesulfonyl)imide dissolved in mixed solution of 1,3‐dioxolane and 1,2‐dimethoxyethane (v/v = 1:1), which was used for major test otherwise stated. 1 wt% of lithium nitrate (LiNO_3_) was blended into the routine electrolyte only for comparison. 20 μL electrolyte was added to the cell. The coin‐type cells were tested in galvanostatic mode within a voltage range of 1.7–2.8 V using a Neware multichannel battery cycler. The electrochemical impedance spectroscopy measurements were performed on a Solartron 1470E electrochemical workstation.

## Supporting information

As a service to our authors and readers, this journal provides supporting information supplied by the authors. Such materials are peer reviewed and may be re‐organized for online delivery, but are not copy‐edited or typeset. Technical support issues arising from supporting information (other than missing files) should be addressed to the authors.

Supplementary
