# Supplementary material for: Janus Separator of Polypropylene‐Supported Cellular Graphene Framework for Sulfur Cathodes with High Utilization in Lithium–Sulfur Batteries
Source: Adv Sci (Weinh). 2015 Oct 1;3(1):1500268. doi: 10.1002/advs.201500268 (PMC5054863; doi:10.1002/advs.201500268)
Supplement: Supplementary file 1 — Supplementary [file ADVS-3-0h-s001.pdf]

Copyright WILEY-VCH Verlag GmbH & Co. KGaA, 69469 Weinheim, Germany, 2015.

ADVANCED SCIENCE
Open Access

Supporting Information

for *Adv. Sci.*, DOI: 10.1002/advs.201500268

Janus Separator of Polypropylene-Supported Cellular Graphene Framework for Sulfur Cathodes with High Utilization in Lithium–Sulfur Batteries

Hong-Jie Peng, Dai-Wei Wang, Jia-Qi Huang, Xin-Bing Cheng, Zhe Yuan, Fei Wei, and Qiang Zhang**

Supporting Information for

Janus Separator of Polypropylene-Supported Cellular Graphene Framework for Sulfur Cathodes with High Utilization in Lithium-Sulfur Bhatteries

Hong-Jie Peng,[+] *Dai-Wei Wang,*[+] *Jia-Qi Huang,** *Xin-Bing Cheng, Zhe Yuan, Fei Wei, Qiang Zhang**

H. J. Peng, D. W. Wang, Dr. J. Q. Huang, X. B. Cheng, Z. Yuan, Prof. F. Wei, Prof. Q. Zhang
Beijing Key Laboratory of Green Chemical Reaction Engineering and Technology
Department of Chemical Engineering
Tsinghua University
Beijing 100084, China
E-mail: jqhuang@tsinghua.edu.cn (J. Q. Huang)
zhang-qiang@mails.tsinghua.edu.cn (Q. Zhang)
[+] H. J. Peng and D. W. Wang contributed equally to this work.

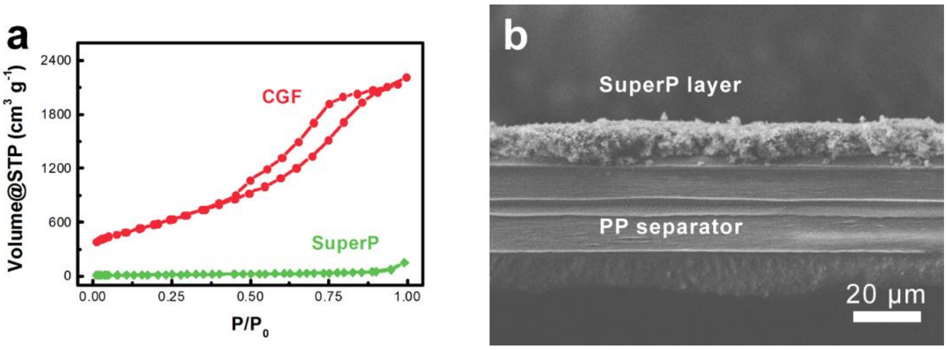

Figure S1. (a) N_2 isotherm of SuperP and (b) cross-sectional SEM image of SuperP separators.

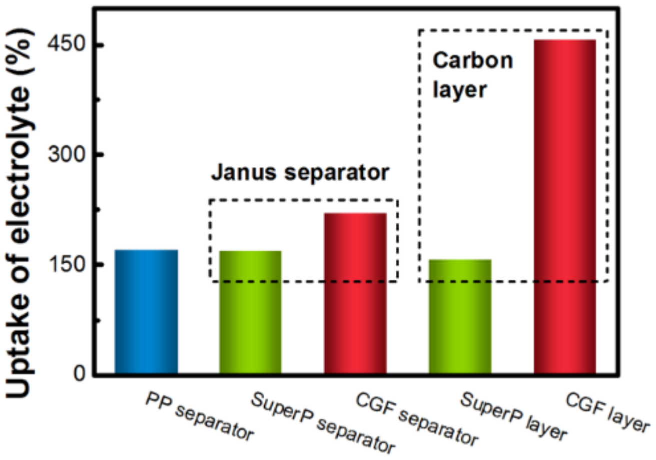

Figure S2. Uptake of electrolyte of PP, SuperP, and CGF, separators as well as SuperP and CCF layers. The uptake of electrolyte of various separators was obtained by weighing the separators before and after dipping into electrolytes for 10 min in an Ar-filled glove box. After dipping, the wet separators were held until no electrolyte dripped, and then were weighed. The uptake of electrolyte was calculated as $(W_{after} - W_{before})/W_{after} \times 100$ %.

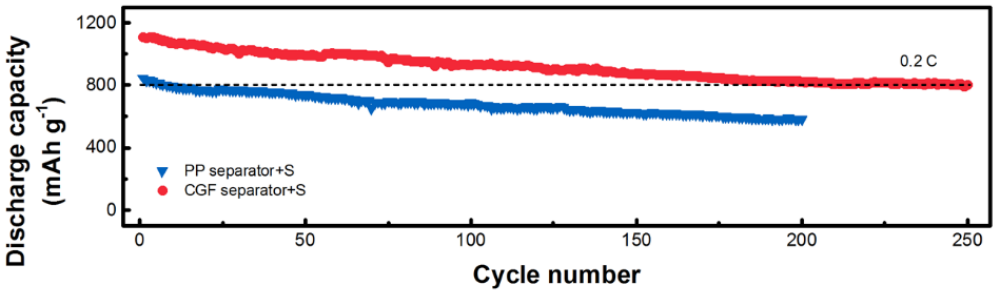

Figure S3. Long-term cycling performance of Li-S cells with PP and CGF separator at 0.2 C.

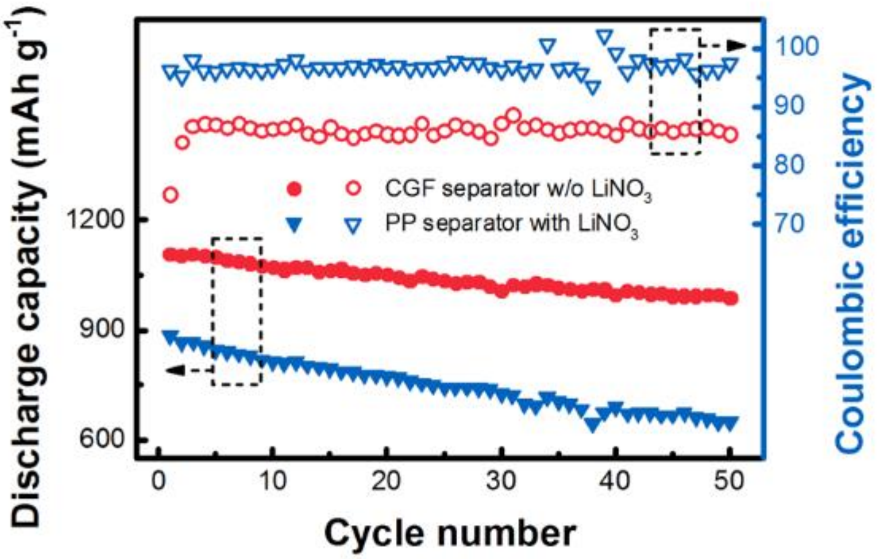

Figure S4. Comparison on cycling performance of Li-S batteries with unprotected lithium/CGF separator/sulfur cathode and $LiNO_3$-protected lithium/PP separator/sulfur cathode.

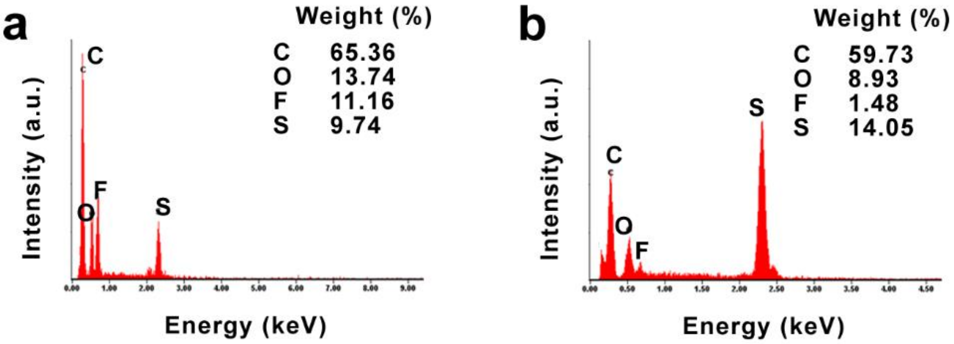

Figure S5. EDS results of cycled separators of anode side: (a) CGF separator and (b) PP separator.

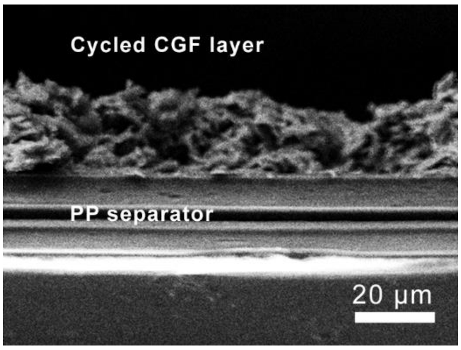

Figure S6. Cross-sectional SEM image of CGF separator after 50 cycles at 0.5 C.

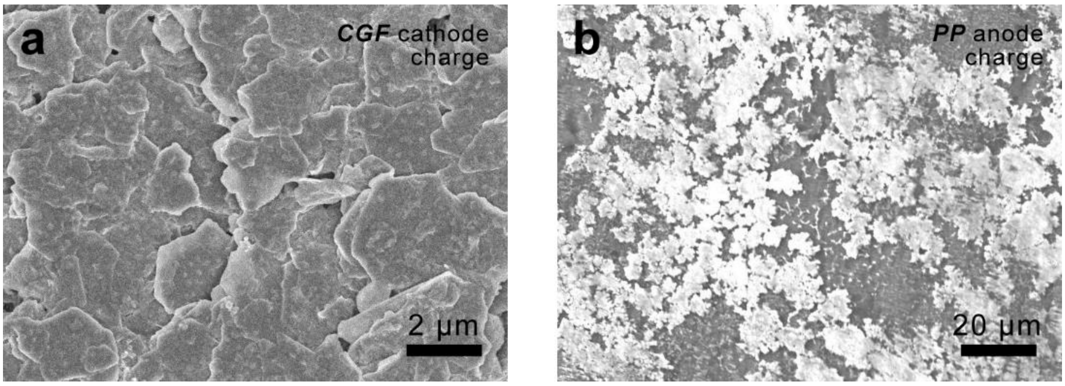

Figure S7. SEM images of (a) cycled CGF separator of cathode side and (b) cycled PP separator of anode side both at charged state after 50 cycles.

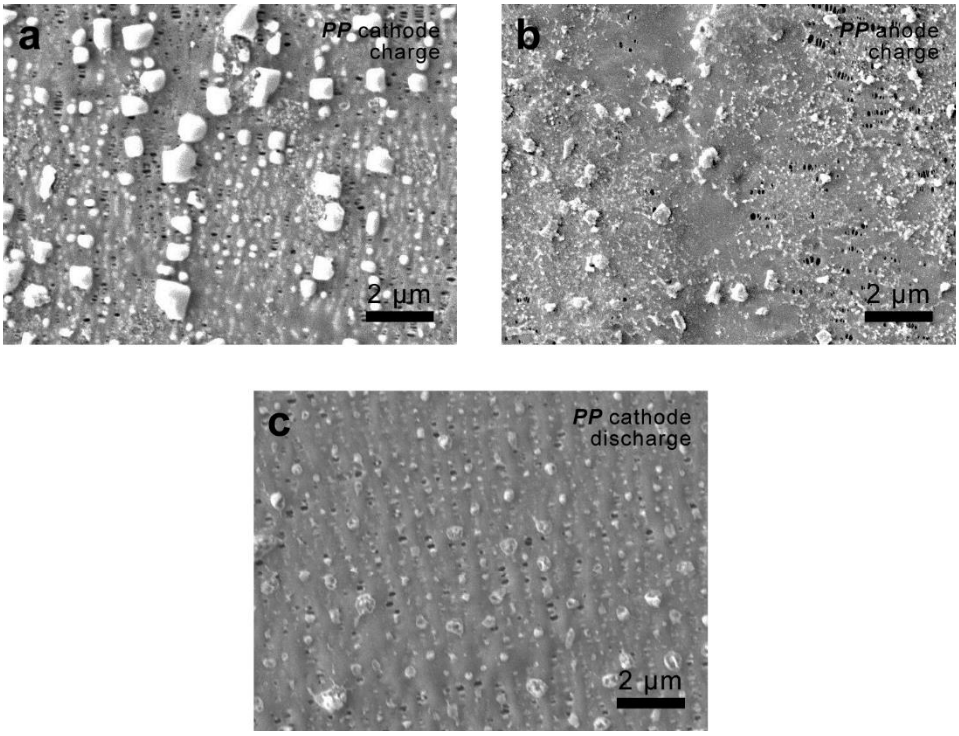

Figure S8. Magnifications of SEM images of cycled PP separators after 5 cycles: (a) cathode side, (b) anode side at charged state, and (c) magnification of “white patches” in **Figure 4j**, showing clogged pores.

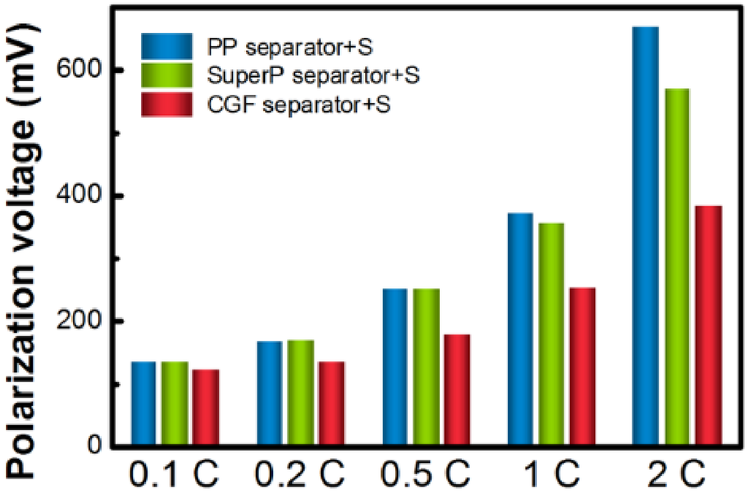

Figure S9. Polarization voltages of sulfur cathodes with PP separator, SuperP separator, and CGF separator at different charge/discharge current densities. The polarization voltages were collected at the depth-of-discharge of 50 %.

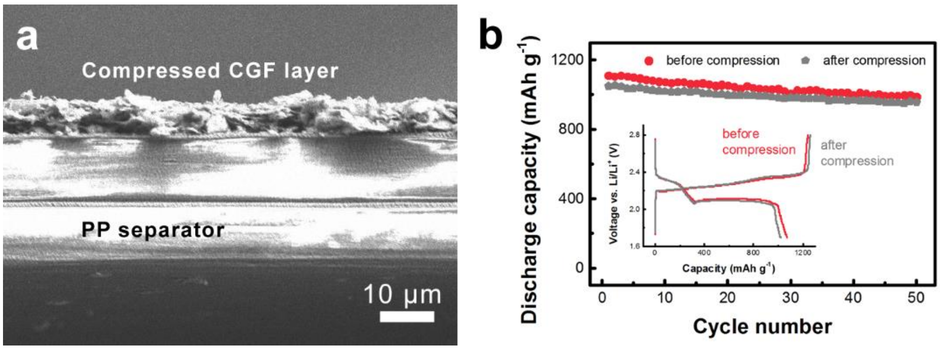

Figure S10. Compressed CGF separator: (a) cross-sectional SEM image and (b) cycling performance of CGF separator before/after compression at a pressure of 10 MPa.

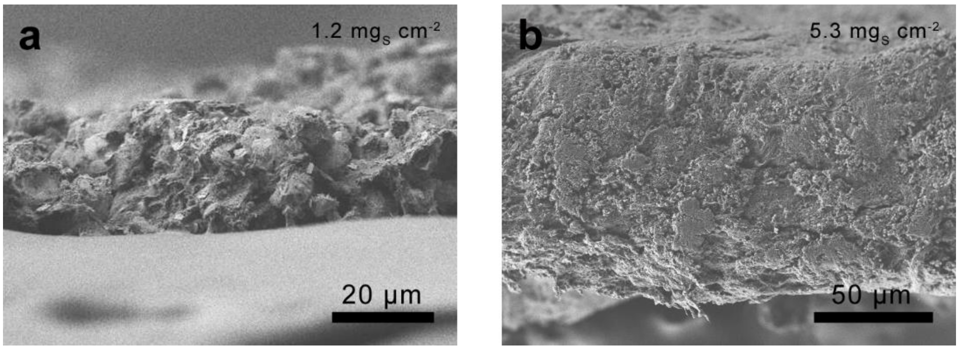

Figure S11. Cross-sectional SEM images of (a) blade-coated sulfur cathode (1.2 mg_S cm^{-2}) and (b) high-sulfur-loading sulfur cathode (5.3 mg_S cm^{-2}).

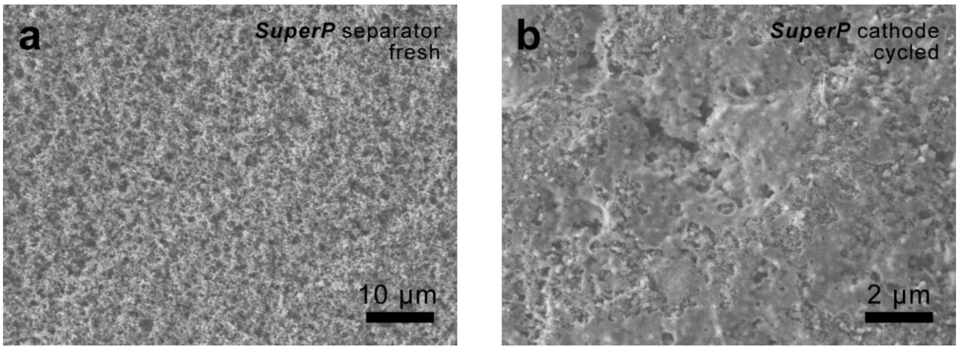

Figure S12. SEM images of surface of (a) SuperP-coated separator and (b) sulfur cathode with SuperP-coated separator after 50 cycles.

Table S1. Volumetric energy densities of lithium-sulfur batteries with different separators.

Cathode		Blade-coated sulfur cathodes			High-sulfur-loading paper electrodes		
Areal loading amount of sulfur (mg cm^{-2})		1.20			5.31		
Separator		PP	CGF	Compressed CGF	PP	CGF	Compressed CGF
Gravimetric capacity (mAh g^{-1})		846	1109	1049	467	1051	949
Areal capacity (mAh cm^{-2})		1.02	1.33	1.26	2.48	5.58	5.04
Thickness (μm)	Cathode	25	25	25	100	100	100
	CGF	0	30	6	0	30	6
	PP	25	25	25	25	25	25
Volumetric energy density[ab] (Wh L^{-1})	Cathode	877	1144	1084	533	1200	1084
	Cathode + CGF	877	520	874	533	923	1022
	Cathode + CGF + PP	439	357	484	427	774	827

a. The volumetric energy density is calculated as follows:

$$ED_V = \frac{C_A \times V}{d}$$

where ED_V, C_A, V, and d are volumetric energy density, areal capacity, average voltage (herein 2.15 V), and total thickness, respectively.

b. The calculated volumetric energy density is underestimated because the thicknesses of cathode and separator in working coin cells, which were compressed under 10 MPa during cell assembly, are smaller than the value applied in calculation.
